# Psychoactive substance use among Tunisian medical students and its impact on academic performance

**DOI:** 10.1192/j.eurpsy.2026.10890

**Published:** 2026-07-31

**Authors:** C. Sridi, M. Aribi, L. Lassoued, F. Chelly, N. Belhadj, A. Fki, M. Maoua

**Affiliations:** 1 Occupational Medicine Department, Sahloul University Hospital; 2 University of Sousse, Faculty of Medicine of Sousse; 3 Research Laboratory LR19SP03; 4 Gynecology and Obstetrics Department, Farhat Hached University Hospital, Sousse, Tunisia

## Abstract

**Introduction:**

Psychoactive substance use (PSU) is a growing concern among university students, particularly those in medical schools who face high academic and emotional demands. Peer influence, stress, and lifestyle factors contribute significantly to this behavior. Such use may impair mental health, cognitive function, and academic achievement.

**Objectives:**

This study aimed to assess the prevalence, and the impact of psychoactive substance use among medical students at the Faculty of Medicine of Sousse (FMSo), Tunisia.

**Methods:**

A cross-sectional analytical study was conducted among FMSo students during the academic year 2023-2024. Data were collected via an online questionnaire that assessed sociodemographic characteristics, lifestyle habits (such as physical activity), and psychoactive substance use (tobacco, alcohol, illicit drugs). Academic performance was measured using self-reported overall grade averages. Univariate and multivariate analyses were performed to determine the association between PSU and academic performance.

**Results:**

Among 1,529 eligible students, 701 completed the survey (response rate: 45.8%), with a female majority (69%). The median age was 22 years [IQR: 21–24]. Most participants came from urban areas (60.2%) and were single (94.2%). Seventy percent reported engaging in physical activity (either occasionally or regularly). Most of the students (47.4%) achieved moderate performance (12-13.99/20). The overall prevalence of PSU among students was 28.4%, including 19.6% for tobacco, 5.4% for alcohol, and 3.4% for illicit drugs. PSU was significantly associated with poor academic performance (p < 0.001). Tobacco smoking also showed a significant negative impact on academic success (p < 0.001). In the final multivariate model, PSU remained an independent predictor of lower academic performance (β = - 1.597, p < 0.001).

**Conclusions:**

Psychoactive substance use is independently associated with reduced academic performance among medical students. Comprehensive prevention strategies focusing on stress reduction, mental health support, and healthy lifestyle promotion are essential to improve students’ well-being and academic success.

**Disclosure of Interest:**

None Declared

